# Changing landscape of medical conferences: identifying the goals motivating virtual vs in-person participation

**DOI:** 10.5116/ijme.676f.ce30

**Published:** 2025-01-30

**Authors:** Sai S. Ram, Daniel Stricker, Carine Pannetier, Nathalie Tabin, Richard W. Costello, Daiana Stolz, Kevin W. Eva, Sören Huwendiek

**Affiliations:** 1 Institute for Medical Education, Department for Assessment and Evaluation, University of Bern, Bern, Switzerland; 2 European Respiratory Society, Lausanne, Switzerland; 3 Department of Respiratory Medicine, Royal College of Surgeons, Dublin, Ireland; 4 The Clinics of Respiratory Medicine and Pulmonary Cell Research, University Hospital Basel, Basel, Switzerland; 5 Centre for Health Education Scholarship, University of British Columbia, Vancouver, Canada

**Keywords:** Conference, motivations, in-person conferences, virtual conferences, hybrid conferences

## Abstract

**Objectives:**

This study was aimed at improving clarity regarding the goals underlying motivation for attendance at international meetings to accommodate evolving needs.

**Methods:**

We performed a case study of a large international medical conference by undertaking (a) semi-structured interviews with 13 multi-disciplinary stakeholders, which underwent thematic analysis, and (b) surveys of 1229 conference attendees, which underwent descriptive statistical analysis and directed content analysis.

**Results:**

Interviews suggested scientific updates and networking are priorities for in-person formats whereas flexibility and reduced travel are priorities for virtual formats. Surveys suggested motivations for attending both in-person and virtual conferences included: scientific updates (81.3% and 85.4%, respectively) and advancements in patient care (76.6%, 78.2%). Social interaction (e.g., to meet experts 80.6% and make/deepen professional connections 69.3%) was highly rated for in-person meetings, but not virtual meetings (51.0% and 30.8%, respectively). 58.9% of attendees prefer future meetings to be hybrid, including both in-person and virtual formats.

**Conclusions:**

We found a disconnect between attendees’ preferences and recommendations currently put forward as socially responsible in terms of climate, equity and diversity. Meeting organisers may need to educate others about the value and costs involved in hybrid formats. When hybrid formats are possible, our data provide guidance on what to prioritize during in-person components and how to combine those with the benefits of global accessibility and flexibility enabled by virtual technology.

## Introduction

Scholarship and research are known to be social enterprises,^1^ resulting in conference travel having long been desirable for academics seeking networking and information sharing opportunities. Such norms are currently being challenged as climate change and the transmission of disease (particularly during the Covid-19 pandemic) have created worldwide counter-pressures. To maintain connection, many in-person conferences have converted to on-line meetings, in whole or in part, thereby leading the community to re-think the sustainability, inclusiveness, and optimal formatting of academic conferences.^2^ This dynamic situation creates risks of disconnect between what conferences can offer, what the community who attends them needs, and how to best address broader societal challenges. To determine how to move conferences forward in a way that is manageable and effective, motivations for attending in-person and virtual formats need to be documented. Without such information, a vicious circle may be created that can impede scientific communication and, in turn, progress: that is, conference attendance is liable to wane, conference delegates are likely to find their experience suboptimal, and conference organizers will likely struggle to maintain a viable meeting, further reducing motivation for attendance.

In-person conferences have long been an important part of academics’ professional development, as they provide environments for face-to-face interactions, hands-on workshops, and opportunities for networking and socialising in a location with minimal  distractions for an attentive audience.^3^ A recent scoping review identified 8 major domains for measuring continuing medical education conference impact and attendee experience, which included career development, logistics and influences on clinical competence and patient communication. ^4^ However, such in-person events have been criticized for being expensive, requiring a large time investment by attendees and organisers, wasting resources, reinforcing gender and social inequities, and having a large carbon footprint. For example, such large-scale international conferences often require air travel, which is known to be a particularly harmful contributor of oxides to the atmosphere. The global warming effect of these emissions is estimated to be three times that of carbon dioxide alone.^5^ Literature further states that aircrafts are responsible for more than 3 percent of annual global greenhouse gas emissions and contribute to the rapid spread of disease, a problem that was made all too clear by COVID-19.^6^

Virtual conferences, in contrast, may facilitate increased participation by providing access to a wider population, including those in disadvantaged economies.^7^ Enabling asynchronous participation through recorded sessions, it is argued, ensures accessibility and convenience for a more diverse range of attendees given varying job role commitments, time constraints and geographic spread. Virtual conference experiences have generally been reported as satisfactory^8^ with motivations to participate including gains in accessibility, inclusivity, and sustainability compared to in-person formats^9^. That said, fully virtual conferences are often more complex to run given the technical burden of organising streaming platforms for a large population, attempting to cater to various time zones and the inability to re-create opportunities such as live clinical skills sessions. In addition, virtual formats remove many networking opportunities.^10^

As a result, conference organizers find themselves in an untenable position as they are expected now to recreate experiences that emulate the serendipity of an in-person meeting,^11^ while ensuring equitable participation through accessible, affordable, inclusive, and environmentally friendly conferences.^12^,^13^ Given that prior research into motivations for conference attendance tends to focus on the experience or desires of delegates in relation to one conference medium or another (e.g., virtual or in-person), there remains a lack of comprehensive analysis of whether and how virtual and in-person conference formats meet different academic and professional needs. To determine how to maintain the benefits of conferences while evolving practices to ensure they remain manageable and effective, a more direct comparison of motivations is required.

Our previous work began to address this issue by examining in what types of sessions delegates report wanting to participate (e.g., clinical skills demonstrations, expert presentations), revealing strategies for optimizing virtual and in-person components of hybrid meetings.^14^ Here we attempt to broaden that exploration, creating a basis for further innovation, by focusing upon what goals delegates are trying to achieve (e.g., scientific updates, meeting experts).

For that purpose, we focused on a large international virtual medical conference, the European Respiratory Society’s virtual congress 2020, which had over 20,000 international multi-disciplinary attendees. More specifically, our three research questions were as follows:

     · What motivates attendance at a large international virtual medical conference versus in-person conferences, from the perspectives of attendees and conference organisers?

     · How well are goals for virtual conference attendance fulfilled? What supports fulfilment of motivations and what barriers get in the way?

     · How satisfied are participants with virtual formats? How could virtual conferences be improved? And what preferences exist for future conference formats?

By investigating the above, our study will provide insights into how to shape future conferences to better serve the professional development needs of attendees.

## Methods

### Overview of Study Design

Using a case study approach,^15^ we gathered data from a variety of sources that focus on a particular situation^16^ - a large, virtually delivered, medical conference – to address our research questions. Interviews with conference organisers informed survey development and a survey was then delivered to a wider sample of conference attendees with closed questions asked to ease comparison across conference formats - in-person versus virtual; open free-text questions were also asked to gain a more descriptive account of respondents’ viewpoints.

#### Context

The case setting was the first virtual European Respiratory Society (ERS) annual congress. The conference attracts individuals with an interest in respiratory medicine from a variety of disciplines and career stages, coming together to present and discuss the latest scientific and clinical advances in the field. Traditionally, this international medical conference has been run “in-person” (i.e., with all delegates gathered at one site) with a program consisting of expert presentations, clinical skills and development opportunities, and networking. From 2016 to 2019, in-person conference attendance averaged n=22,422. When the COVID-19 pandemic emerged, the 2020 meeting was moved to a virtual format and enrolled n=29,020 attendees. That meeting included a live online streaming platform that was structured similar to news channels (i.e., attendees could stream a variety of “programmes”) that included presentations delivered by the world’s respiratory experts to enable discussion of the latest scientific and clinical advances across the field of respiratory medicine. The main conference programme was conducted during working hours of Central European Summer Time, but included a 24-hour stream of sessions to cater to other countries. In addition to providing knowledge updates, clinical debates and case discussions were encouraged. The study was deemed exempt from ethical review by the Regional Ethics Committee of the Canton of Bern because no health-related personal data or biological material were used. After gaining informed consent from participants, confidentiality and anonymity of all survey data and speaker interview data were maintained throughout the study, including removal of identifying information from quotations.

#### Stakeholder Interviews

To understand the variety of motivations that organisers perceive to influence delegates’ attendance and to develop survey items, we conducted semi-structured interviews with ERS stakeholders. Purposive sampling was used by virtue of attempting to recruit individuals who had extensive conference organising experience. To that end, we approached all 23 people who held specific functions within the ERS society (e.g., Chair of a committee) via e-mail requesting their participation. An interview guidewas created that questioned participants’ professional role and experience, perceptions of motivations for conference attendance, and how perceptions were expected to change with the transition to a virtual conference format.

### Data collection

Interviews took place over Zoom, were audio‐recorded, and transcribed verbatim (apart from removal of any identifiable information) by SR. Verbal consent was obtained prior to the interview date and asked for again immediately before the start of the interview commencement. We strove for transferability by inviting participants to speak about their conference experience broadly rather than feeling constrained to speak specifically about the ERS annual congress; we strove to ensure dependability by continuing data collection until no new themes emerged through iterative data analysis.^17^ In all, interviews with 13 stakeholders were conducted, which lasted 33 minutes on average. Seven countries and nine different professional roles/specialties were represented*. *After every interview, analytic memos were written and SR listened to the recordings and highlighted any interesting aspects. These were discussed, and modifications to the interview guide were made as necessary to delve deeper into stakeholder perspectives, in consultation with SH. The final interview guide is published elsewherebecause it was also used as part of another study^18^ focused on different research questions (investigating whether subgroups of conference attendees can be meaningfully identified).

### Data analysis

After transcription, a six-phase thematic analysis approach,^19^ was applied to the data. This included (1) familiarisation with data (2) generating initial codes (3) searching for themes (4) reviewing themes (5) defining themes and (6) creating a narrative. After generating an initial set of codes, themes were developed and finalised in cycles of feedback and discussion with the research group.

#### Reflexivity

Both emic (within the setting) and etic (outside the setting) perspectives of our research team were considered. DSto, RC, NT, CP are organizers for the ERS and, hence, represented emic perspectives (within the setting). PhD student SR attended the first virtual ERS conference and kept a reflective diary. SH and KE are medical education researchers with extensive conference experience. SR, SH and KE represented the etic (outside the setting) perspective.  To limit the extent to which preconceptions overly influenced data collection or analysis, the research team had repeated discussions throughout the various study stages.

#### Survey of Attendees

In an effort to optimize the credibility of our observations through triangulation, we also gathered data from a different source (conference delegates) using different data collection methods (surveys). A guide for survey creation was followed.^20^ We undertook: (1) literature review of relevant research on conference motivations; (2) interviews with the ERS stakeholders, as specified above; (3) comparison of the information gleaned from both sources to create a preliminary survey that was sent to experts for their feedback (N=7: 4 individuals who organise large-scale international conferences, 1 psychologist, and 2 medical education experts); (4) survey modification and creation within an online tool; and (5) cognitive interviews with members of the target population (N=3) to ensure that the survey was understood as intended. Efforts to ensure content validity evidence were supported by stages (1) (2) and (3); response process validity was supported by stage (5).

The final survey, which is already published^18^, had a total of 27 questions. Demographic variables that were collected included age, gender, country and workplace of practice and professional role. To ascertain attendee preferences, participants rated a list of 15 distinct conference attendance motivations (using 7-point Likert scales ranging from 1=strongly disagree to 7=strongly agree), drawn from the literature and stakeholder interviews. For this study, questions were asked about why they usually attend the ERS congress and why they chose to attend the first virtual conference. Open free-text questions were included to understand their motivations and what facilitators or barriers impacted upon their fulfilment.

### Data collection

An online tool, SurveyMonkey, was used to create and deliver the survey to all attendees of the ERS virtual congress 2020. A link was sent via email, after the conference, with a request to help shape future conferences through their participation. Within the invitation e-mail, it was explicitly stated that by filling in the survey participants gave their informed consent and that any questions about the study could be sent to the researchers. No identifying information was collected. Within the first month of the survey being disseminated, two reminder emails were sent and attendees were notified of an incentive to win a free registration to the ERS Congress 2021. The initial survey was sent to 29,020 attendees. 1,229 individuals, 4.2% of conference attendees, completed the introductory portion of the survey and 75% of them (N=915) responded to at least 2/3rds of all questions.

### Data analysis

Descriptive analysis was conducted for the demographic data and motivations for in-person and virtual conference attendance. While the extensive development work derived above was undertaken to ensure the survey was fit for purpose, internal consistency (reliability) analyses were not conducted for this study because we were interested in the individual questions and not possible factors of a construct. Free text responses underwent directed content analysis.^21^ Keywords derived from the literature and stakeholder interviews were noted as a starting point for coding the data, with additional codes being added as the analytic process continued. Text that could not be categorised with the initial coding scheme was given a new category with revisions to the coding scheme being conducted iteratively until analysis was complete. The content analysis was a continual process whereby initial and final categories were all revised and refined by the research team as a whole until there was consensus.

## Results

### Stakeholder Interviews

Five main themes were derived from the data: (1) Stakeholders expected motivations for attendance at in-person meetings to be dominantly driven by a desire for scientific updates, interaction with peers and colleagues, and fostering inspiration/enthusiasm within the field; (2) gaining knowledge was expected to be easier in virtual platforms; (3) flexible and convenient access with broadened global participation through reduced travel were expected to be strong motivators for virtual formats; (4) long hours in front of a screen were thought likely to decrease concentration, decrease interaction, and create greater challenge balancing conference attendance with other daily commitments; (5) decreased interaction with others was expected to have a detrimental impact on professional and personal development. Table 1 describes these themes with corresponding quotes.

### Attendees Survey

52% of respondents reported being male and age was normally distributed with a peak in the 41–45-year-old range. The modal workplace was a university hospital (33%, N=477), followed by academic institution (20%, N=288) and university hospital (16%, N=228); any other workplaces were named by <10% of participants. 141 participants did not specify their region, but of those who did, 58% (N=630) attended from Europe, 20% (N=217) attended from Asia, 10% (N=111) attended from South America, 5% (N=58) attended from North America, 4% (N=38) attended from Oceania, and 3% (N=34) attended from Africa. These numbers indicate slight under-representation of Europeans relative to the full pool of attendees: for the total number of participants who attended the virtual ERS Congress 2020, 71% (N=5620) attended from Europe, 11% (N=912) attended from Asia, 9% (N=676) attended from South America, 4% (N=314) attended from North America, 3% (N=226) attended from Oceania, and 2% (N=165) attended from Africa.

#### Motivations for attendance at virtual conferences versus in-person conference attendance

From the closed questions, the percentage of respondents who agreed or strongly agreed is illustrated in Table 2.

#### Fulfilment of attendees’ motivations for virtual conference attendance

The motivations for virtual attendance (see Table 3) that were most frequently rated as fulfilled included: to learn the latest scientific updates (72.7%, N=736), to learn the latest advancements in patient care (64.7%, N=652) and to support my career development (50.4%, N=512).

The motivations for virtual attendance that were least frequently rated as fulfilled included: to present my scientific/academic work (25.2%, N=255), to make/deepen professional connections (25.2%, N=255) and to interact and spend time with peers (14.9%, N=151).

#### Factors that supported or created barriers to fulfilment of attendees' motivations

From the open-ended questions posed on the survey, the three most frequently mentioned aspects of virtual conference attendance reported as fulfilling attendees' motivations and the five most frequently mentioned barriers are shown in Table 4. Participant responses that were named by >10% of total participants are included in the table.

**Table 1:** Themes and corresponding quotes for what stakeholders expected regarding in-person and virtual format motivations for attendance

Themes	Corresponding Quote
**Theme 1:** The main motivations underlying attendees’ desire to attend in-person conferences were scientific updates, interaction with peers and colleagues, and fostering inspiration/enthusiasm in an academic field. Description: Stakeholders believed that scientific updates are the main factor motivating in-person conference attendance with latest advancements and new knowledge being highlighted. Noted alongside this theme was the value of networking, meeting other people, forming new collaborative links and, for younger members, joining and integrating into the community. Personal encouragement of an individual’s career or inspiration to implement changes and ideas for new avenues of others were also expected to be fostered.	“The main motivation is, of course, apart from to be updated about the last advances in a specific field, probably the main motivation is to meet people and to create networks of collaborations.” – Stakeholder 7
	“to attract young members and to see ways in order to be more productive and more collaborative. This is the general idea and then we have to find ways to focus on new [scientific] developments” – Stakeholder 3
	“I think these colleagues are mostly chest physicians, pulmonologists, interested maybe in certain aspects of respiratory medicine but they only consume what’s new, so they come to the, in your congresses to obtain an update in their fields of interests. […] it’s a huge chance to communicate, to interact with your peers and to meet and congress to plan your new projects.”- Stakeholder 8
	“it’s gaining a boost in your enthusiasm for your profession. I think an important role for the conferences is first getting enthusiastic again, hearing new things and meeting other people, that is very important aspect of conferences as well” – Stakeholder 2
**Theme 2: ** Gaining knowledge was expected to be easier in virtual platforms. Description: The virtual format was expected to allow viewing of pre-recorded and live recorded sessions given the ease of switching to and from parallel sessions. Having all the scientific information on one platform makes it easier to share all content with the audience.	“everything is recorded or even pre-recorded and then obviously you can go to all the parallel sessions if you want, because you can’t do that in the real congress”- Stakeholder 12
	“sharing the scientific information will also be more possible now of course and an advantage of the digital format”- Stakeholder 5
**Theme 3:** Flexible and convenient access with broadened global participation through reduced travel were expected to be strong motivators for virtual formats. Description: The virtual format was expected to allow attendees to stream without travel cost and time burdens. Accessing virtual recordings makes it easier for attendees to accommodate their personal and work schedules.	“it’s just like cherry picking, we’ve picked that talks and you don’t have to sit there for all session. You can do it at any time, whenever you want, so I think that’s a huge benefit” – Stakeholder 2
	“in one way it’s good because you do not have to travel, you do not have to move, you can do it in a fractioned way, for example one evening and then another piece in another evening, maybe also when you have spare time.” – Stakeholder 5
**Theme 4:** Long hours in front of the screen were thought likely to decrease concentration, decrease interaction and create challenges balancing other daily commitments. Description: Virtual formats would mean spending hours of time in front of a screen, which can lead to decreased engagement and concentration. Physically being in a different location, city or travelling could mean that other day to day tasks such as work commitments or family commitments, if joining from home, do not interfere with the time dedicated to in-person conferences. For speakers, having the audience in-person was expected to change the ambience whereas speaking to a screen can be difficult.	“Online you can lose some attention because of course you’re doing something else, usually, you’re also, you know, you have the kids or you have someone who’s coming in and so you can lose attention and then you do not see it in person who’s speaking” – Stakeholder 11
	“I mean in big auditoria of course it’s a different thing, also the [virtual] interaction with the crowd is limited, maybe you have some multiple-choice questions or some votes or some polls, but especially in the abstracts it’s not the same as when you can really see your audience and that interaction I think will be missing, for sure.” – Stakeholder 6
**Theme 5: **Decreased interaction with other delegates was expected to have a detrimental impact on professional and personal development. Description: Using the example of poster discussions, live discussion with members gathered around a poster was thought to be very different relative to online interactions. The live atmosphere was expected to foster spontaneous meetings and discussions while allowing new young researchers to be introduced into the community. Personal development was also expected to decline by virtue of lessened connection with friends.	“I’ll see how it goes with the posters, because I think the posters discussions that we have, around the posters and going around meeting people is all part of the networking and talking and introducing your new young researches to a broader group. I’m not sure how that’s going to work.” – Stakeholder 1
	“if you don’t know people and you have to be quite careful and we’re not able to create this personal linkage, I think it’s very difficult to advance and the all thing it becomes so dry and uninteresting […] without having this personal attachment because, you know, when you live in the hospital, when your life is 98% medicine.” – Stakeholder 9

**Table 2:** Motivations for attendance at virtual conferences and in-person conferences

Conference motivations	Percentage % (and proportion) who agreed or strongly agreed** with each motivator	
	Virtual format	In-person format
to learn the latest scientific findings	85.4 (N=864/1012)	81.3 (N=584/718)
to learn the latest advancements in patient care	78.2 (N=785/1004)	76.6 (N=552/721)
to support my career development	70.2 (N=711/1013)	67.4 (N=480/712)
to meet experts and leaders in the field	51.0 (N=515/1009)	80.6 (N=580/720)*
to fulfil the requirements of professional certification bodies, such as attaining CPD/CME credits	39.8 (N=402/1011)	34.9 (N=250/716)
to improve my teaching skills	37.7 (N=380/1008)	39.3 (N=279/710)
to foster personal change	36.0 (N=363/1008)	42.6 (N=304/714)
to improve my practical clinical skills (e.g., online live bronchoscopy procedure)	35.2 (N=356/1012)	40.8 (N=292/715)
to support career developments of others	32.8 (N=330/1007)	30.1 (N=214/712)
to improve my communication skills	32.6 (N=330/1012)	40.0 (N=287/718)
to make/deepen professional connections	30.8 (N=310/1008)	69.3 (N=498/719)*
to foster change in my workplace	30.8 (N=310/1005)	40.9 (N=292/714)
to present my scientific/academic work	27.5 (N=277/1006)	54.6 (N=392/718)*
to socially interact and spend time with peers	20.6 (N=208/1008)	67.0 (N=480/716)*
to explore the city/region where the conference is held	N/A	46.0 (N=329/715)

* With a sample as large as that included in this study (N=1004 to 1013 for all virtual format questions and N=710 to 721 for all in-person format questions), even small differences appear statistically significant; as such, we concentrate on those differences that are substantial by highlighting motivations with more than a 20% difference between virtual and in-person conference attendance. ** Participants who selected 6 or 7 on Likert scales where 1 strongly disagrees and 7 strongly agree (indicating strong agreement with each factor being a motivator for conference attendance)

**Table 3:** Motivations for virtual conference attendance and the percentage of participants who deemed that motivation to have been fulfilled at the ERS conference.

Virtual motivation	Percentage % (and proportion) of respondents reporting a motivation had been fulfilled*
to learn the latest scientific findings	72.7% (N=736/1012)
to learn the latest advancements in patient care	64.7 (N=652/1007)
to support my career development	50.4 (N=512/1016)
to meet experts and leaders in the field	37.7 (N=379/1006)
to foster personal change	31.9 (N=323/1012)
to fulfil the requirements of professional certification bodies, such as attaining CPD/CME credits	29.4 (N=297/1013)
to improve my teaching skills	28.6 (N=289/1009)
to foster change in my workplace	27.2 (N=275/1009)
to improve my communication skills (e.g., patient communication, team communication)	27.1 (N=275/1012)
to improve my practical clinical skills (e.g., in-person bronchoscopy)	26.6 (N=269/1012)
to support career developments of others	25.9 (N=261/1005)
to make/deepen professional connections	25.2 (N=255/1007)
to present my scientific/academic work	25.2 (N=255/1012)
to socially interact and spend time with peers	14.9 (N=151/1012)

*Participants who selected 6 or 7 on Likert scales where 1 strongly disagrees and 7 strongly agree (indicating strong agreement with each factor being fulfilled for motivation of conference attendance)

**Table 4:** Frequently mentioned factors that supported and created barriers to fulfilment of motivations for virtual conference attendance

Items	Category	Further supporting comments
Aspects that supported fulfilling motivations for virtual conference attendance Total N=1115	Easy accessibility37.7%, N= 420	wider global access virtually less effort needed to attend than in person e.g., less travel costs for flights and accommodation
	Flexible timing28.2%, N= 314	ability to review recorded sessions at a later time amongst work commitments switch easily from congruent sessions
	Online session formats11.1%, N= 124	shorter in duration concise in delivery of key topics
Barriers to fulfilling motivations for virtual conference attendance Total N= 724	Difficultly interacting with others32.5%, N= 235	virtual interaction with speakers, other experts and colleagues perceived as not possible in the same waymissing the impromptu nature of spontaneous in-person meetings e.g., in corridors not being in the same time zone so watching sessions at different times e.g., recordings
	Difficult to network19.5%, N= 141	challenging to interact with other members to form collaborative links challenging to introduce younger members into the scientific community
	Technical issues14.2%, N= 103	technical issues pre-conference when uploading material onto the platform, during the conference when streaming sessions and after when accessing post-conference recordings
	Online formats cause difficulty for extended concentration12,4%, N= 77	reduced concentration when watching online sessions hard to concentrate on the screen
	Allocating time for conference amongst work commitments10.6%, N=90	Busy with clinical work commitments to attend online sessions

* Responses with >10% of total participants are included in the table.

In addition, participants wished to (4) elongate the conference time frame (12.9%, N=103) by, for example, having the conference over a longer time duration and (5) improve interactivity at the overall conference between members (11.5%, N=92), which included improving interactions between participants and speakers.

#### Participants’ future format preference and reasoning

From a closed question with 1,229 responses, the majority of respondents (58.9%, N=724) prefer a combined in-person and virtual (i.e., hybrid) format – a conference format which allows live online streaming of the in-person conference and may include additional virtual sessions. By comparison, 22.9% (N=281) prefer for future meetings to be held in-person and 16.3% (N=200) prefer virtual formats.

There were, overall, 1146 free text comments regarding future format preferences. 38.4% (N=440) suggested hybrid conferences were preferred because they combine the advantages of both meeting formats. In-person conferences were perceived as allowing better interactivity with other delegates (24.0%, N=275), better networking opportunities (6.0%, N=69), better concentration (3.3%, N=38), the opportunity to explore location (1.1%, N=12) by taking advantage of the conference being in a new city, and personal preference (0.8%, N=9). Virtual conference formats were preferred for being time saving (13.5%, N=155), cheaper (6.7%, N=77), safer during the pandemic (3.8%, N=43) and more globally inclusive (2.4%, N=28). N=83 did not provide a reason for their format preference.

## Discussion

Our study has shown that motivations for conference attendance are variable and differ between in-person and virtual formats, yielding a variety of observations that are valuable for planning future meetings. Adding to our previous work, with a different sample of conference delegates, exploring what types of sessions they value,^14^ the motivations in this study offer an abstraction of the goals delegates are striving to achieve, thereby providing a means of guiding innovative efforts to design or update conference programs. Our previous work with this particular sample revealed that conference delegates should not be considered to be a uniform group – 3 distinct groups, in fact, were identified based on differences in their motivations for attendance.^18^ That said, common to all, was motivation for learning, thereby stimulating a deeper exploration into what particular aspects of learning might best be fulfilled by in-person vs virtual formats. In the following paragraphs we highlight three specific findings for their capacity to add to existing literature regarding conference planning: (1) Scientific updates are the main motivator for attendance regardless of which format (virtual or in-person) is used; (2) interaction within virtual formats was not found to be fulfilled, but it is also not a highly rated motivator for attendance at virtual meetings; and, (3) the majority of attendees would like hybrid formats with a combination of in-person and virtual components for future conferences.

As noted, high ratings were assigned on our survey to scientific updates in both virtual and in-person formats. Further, attendees’ free text comments indicated that motivations to update one’s knowledge through virtual formats are likely best fulfilled by holding shorter online sessions. Stakeholders and attendees expected and reported, respectively, that long virtual sessions could hinder concentration. Literature does not explore this within conference settings but there is suggestion that shorter talks reduce the cognitive load imposed by attention span issues when content is presented via video.^21^ In addition, the pause and rewind function of asynchronous e-learning has been reported as helping attendees self-manage learning remotely.^22^ This all suggests that delegates wish for conference organisers to prolong the length of time over which conference materials are available to enable greater learning. For example, by using a virtual platform for increased access to record materials throughout the year (and in various languages to promote equity, diversity, and inclusion, if possible).

Second, it is noteworthy that highly rated motivations for attendance at in-person meetings included social interactions (e.g., *meet experts* and make*/deepen professional connections) *thatwere less highly rated for attendance at virtual meetings. This basic observation is consistent with pre-existing literature revealing that social interactions are best delivered in-person^23^ and that the quality of engagement and communication within virtual conferences has been criticized.^24^ Where our study adds to such findings is with respect to fulfilment of motivations and what barriers prevent motivation fulfilment. Interaction within virtual conferences was not a main motivator for attendance, suggesting that those who attend such meetings are unlikely to be disappointed by the lack of opportunity to interact, thus freeing organisers to focus resources on what attendees most anticipate. In fact, efforts to build interaction into the virtual ERS meeting were not particularly well received. Stakeholder interviews indicated that they expected decreased virtual interaction and that such declines could have a detrimental impact on professional and personal development. Specifically, they noted that poster sessions and live discussion with other delegates were likely to be very different online. As a way to target this anticipated challenge, conference organisers created online poster sessions that included expert facilitators to encourage interaction. Written free text responses from survey respondents, however, suggested that such virtual sessions still lacked the same sense of interaction and did not fulfil attendees’ needs. Given survey respondents’ indications that strong opportunities for interaction are not expected in virtual meetings these efforts and resources might have been better spent in other ways.

Finally, as hybrid conferences were preferred for future events in our study, it will be important for meeting planners to consider how and when to prioritize which medium. Such preferences suggest that those concerned with the negative environmental impact of travel^2^ or with social inequities created by in-person components of meetings (due to financial, physical, or social constraints) have work to do to educate the general population of delegates regarding the value of virtual conference formats. Those who wish to prioritize attendee preferences by holding a hybrid conference, in contrast, need to recognize that virtual attendees do not have the same experience as in-person attendees. They cannot interact with those attending in person in the same capacity; they may not have the chance to ask and receive questions; and, they do not have access to formal and informal gatherings outside of standard sessions. If hybrid conferences are available, attendees can choose to attend in a way that best suits their individual needs, but our findings suggest that delegates would benefit from organizers prioritizing social activity during in-person components and information delivery online. Participants in previous research offered guidance in that regard, suggesting that clinical skills sessions and expert presentations be the primary focus for in-person components while the latter can be better combined with case discussions and clinical updates virtually.^13^ In any case, the more the conference experience can be adaptable, with more flexible and dynamic use of content, the more it can be modulated to the needs of each attendee.^10^

In summary, our study can be used to provide guidance to conference organisers who strive to combine the interactivity benefits of in-person meetings with the flexibility of virtual meetings. Strengths of the work include contributing new knowledge by delving deeper into the goals underlying motivations for both in-person and virtual conferences and by juxtaposing expectations with motivations in a way that provides more guidance regarding the differences between formats (tempering concern about the inability to meet all academic needs within each type of meeting). Improvements may be made, as a result, to virtual, in-person, or hybrid formats by prioritizing resources towards the activity and goals that are most suitable and most drive delegates to one conference format or another. Combining interviews and surveys for such a broadscale investigation into attendees’ views and stakeholders’ perspectives has not been done previously.

Limitations of this study include the low response rate of the survey, but the extent to which that is an issue is lessened by virtue of the overall number of responses being high and respondents seeming largely representative of conference attendees. Further, the results generated might be limited by selection bias as participants became eligible only by virtue of having chosen to attend the virtual conference. It is important to remember, also, that this study is a snapshot of early virtual conference research and, thus, may change over time as people gain more experience and comfort interacting in the context of innovative meeting formats. Future research may include more detailed investigation of what components attendees would like to see during in-person versus virtual components of hybrid formats.

## Conclusions

Delegates’ goals underlying their motivations for conference attendance differ between in-person and virtual conference attendance. Hybrid conferences were the preferred future format, suggesting that meeting organizers need either to educate others about the value and costs involved in non-hybrid formats or, when hybrid formats are possible, they would do well to prioritise means of finding innovative ways for strengthening interactivity during in-person components (e.g., networking opportunities) while combining those with the benefits of global accessibility and flexibility enabled by virtual technology. When such formats are not possible, our findings suggest that conference strategy should be catered to attendee expectations and motivations without assuming those to be the same in both in-person and virtual formats.

### Acknowledgements

We would like to thank the participants in this study and the European Respiratory Society for funding this study as part of a PhD research project.

### Conflict of Interest

The study was part of SR’s PhD project, which is sponsored by the ERS. No other authors have competing interests.
